# High USP32 expression contributes to cancer progression and is correlated with immune infiltrates in hepatocellular carcinoma

**DOI:** 10.1186/s12885-023-11617-4

**Published:** 2023-11-13

**Authors:** Mengxi Xiu, Wenfang Bao, Jialin Wang, Jingde Chen, Yandong Li, Yanan Hai

**Affiliations:** Department of Oncology, Shanghai East Hospital, Tongji University School of Medicine, Shanghai, 200120 China

**Keywords:** Hepatocellular carcinoma, USP32, Immune infiltrates, Tumor environment, Targeted therapy, Immunotherapy

## Abstract

**Background:**

Ubiquitin-specific protease 32 (USP32) is a highly conserved gene that promotes cancer progression. However, its role in hepatocellular carcinoma (HCC) is not well understood. The aim of this project is to explore the clinical significance and functions of USP32 in HCC.

**Methods:**

The expression of USP32 in HCC was evaluated using data from TCGA, GEO, TISCH, tissue microarray, and human HCC samples from our hospital. Survival analysis, PPI analysis and GSEA analysis were performed to evaluate USP32-related clinical significance, key molecules and enrichment pathways. Using the ssGSEA algorithm and TIMER, we investigated the relationships between USP32 and immune infiltrates in the TME. Univariate and multivariate Cox regression analyses were then used to identify key USP32-related immunomodulators and constructed a USP32-related immune prognostic model. Finally, CCK8, transwell and colony formation assays of HCC cells were performed and an HCC nude mouse model was established to verify the oncogenic role of USP32.

**Results:**

USP32 is overexpressed in HCC and its expression is an independent predictive factor for outcomes of HCC patients. USP32 is associated with pathways related to cell behaviors and cancer signaling, and its expression is significantly correlated with the infiltration of immune cells in the TME. We also successfully constructed a USP32-related immune prognostic model using 5 genes. Wet experiments confirmed that knockdown of USP32 could repress the proliferation, colony formation and migration of HCC cells in vitro and inhibit tumor growth in vivo.

**Conclusion:**

USP32 is highly expressed in HCC and closely correlates with the TME of HCC. It is a potential target for improving the efficacy of chemotherapy and developing new strategies for targeted therapy and immunotherapy in HCC.

**Supplementary Information:**

The online version contains supplementary material available at 10.1186/s12885-023-11617-4.

## Introduction

Hepatocellular carcinoma (HCC) is a common malignancy, responsible for over 900,000 new cases and 800,000 deaths annually [1]. Despite the efforts in developing systemic chemotherapy strategies, the overall survival rate of HCC patients remains poor [1, 2]. In recent decades, targeted and immunotherapies have emerged as novel therapeutic methods to alleviate HCC progression and improved the life quality of HCC patients. However, the economic burden of HCC treatment is still high, and patient outcomes remain undesirable [2]. Therefore, further discovery of the underlying molecular mechanisms of HCC is urgently needed to develop new therapeutic strategies and improve patient outcomes.

Ubiquitylation is a posttranslational modification that regulates cell signaling pathways, protein activities, DNA damage responses and intracellular trafficking [3]. Like other posttranslational modifications, cellular ubiquitination can be reversed by deubiquitinating enzymes (DUBs), which remove ubiquitin from substrates and disassemble ubiquitin chains [3]. Ubiquitin-specific proteases (USPs) are the largest and most diverse subgroup of DUBs and target specific protein substrates to regulate gene silencing, cell apoptosis, and immune responses [4]. Recent studies have implicated USPs in the pathogenesis of several human diseases, such as inflammation-related diseases and cancer [4, 5].

**Fig. 1:**
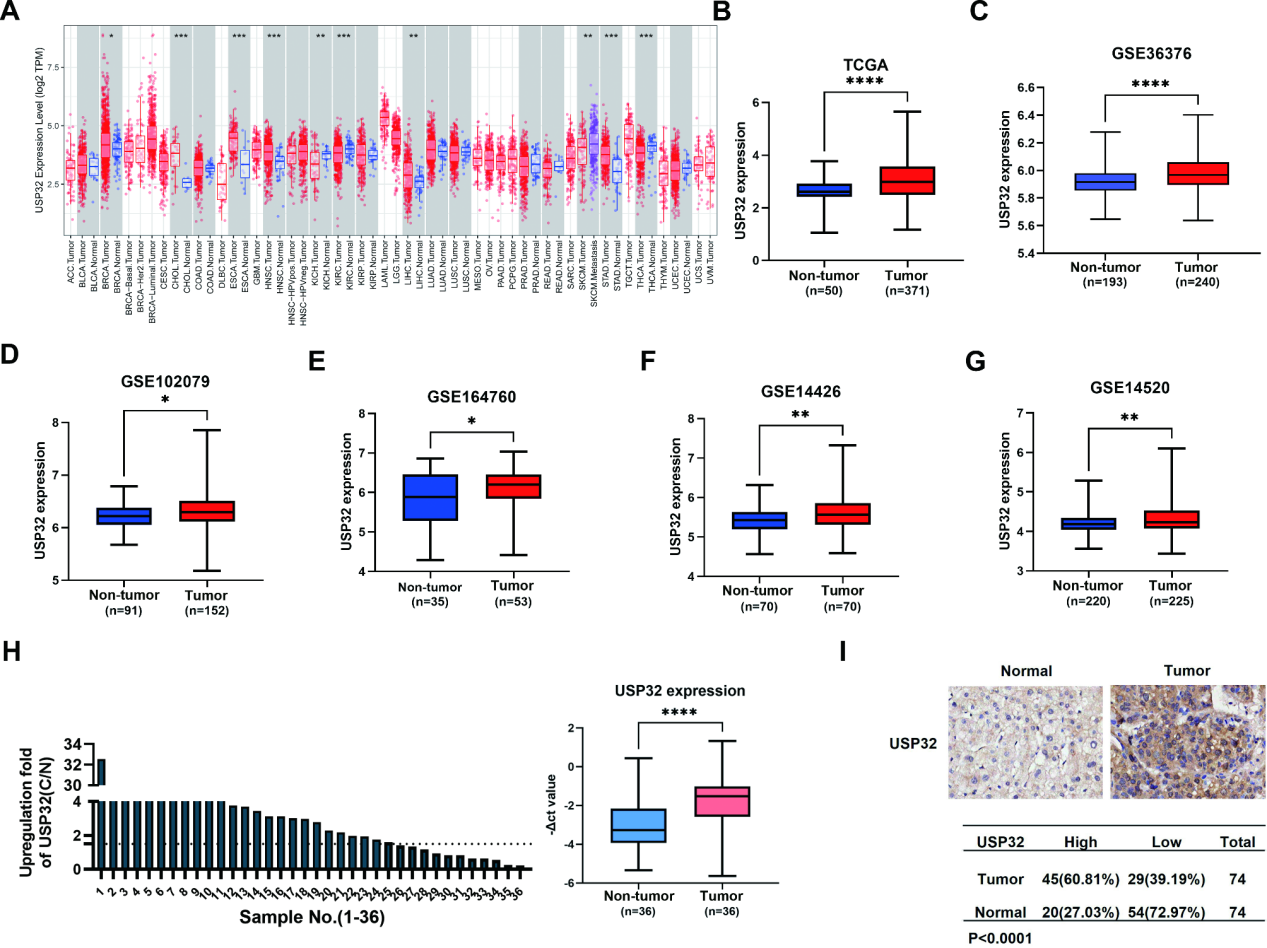
USP32 expression is significantly increased in HCC tissues. (**A**) USP32 expression in different cancer types from TCGA datasets. (**B-G**) Human HCC gene expression data from public TCGA-LIHC, GSE36376, GSE102079, GSE164760, GSE14426 and GSE14520 were analyzed to compare USP32 mRNA levels between tumor and normal tissues. The Manne-Whitney-Wilcox test was used. *P < 0.05, ****P < 0.0001. (**H**) USP32 mRNA expression in 36 pairs of HCC samples and adjacent normal samples were estimated through qRT-PCR analysis, and -ΔCT value represented the relative expression of USP32. The unpaired two-tailed Student’s t-test was utilized. **P < 0.01. (**I**) The USP32 protein level in HCC was detected by IHC analysis on a TMA containing 75 HCC tissues and paired adjacent normal tissues (74 are available for analysis). The *chi-square* test was utilized

USP32 gene localizes on chromosome 17q23 and it is a highly conserved gene. It shares over 90% sequence identity with USP6, the first DUB discovered as an oncogene [6]. In 2010, Akhavantabasi et al. identified that USP32 is a membrane-bound DUB highly expressed in breast cancer (BC), and knockdown of USP32 led to a decrease in the proliferation and migration of BC cells [7]. In subsequent studies, several studies supported that USP32 functions as a universal oncogene in cancer. In 2020, our laboratory first reported that USP32 plays an oncogenic role in gastric carcinoma (GC) through promoting tumor growth, metastasis, and cisplatin resistance [8]. In 2021, USP32 was found to be overexpressed in epithelial ovarian cancer (EOC), particularly in metastatic peritoneal tumors, and it positively regulates the proliferation and epithelial-mesenchymal transition (EMT) capabilities of cancer cells [9]. In 2022 and 2023, researchers confirmed that USP32 act as an oncogene in glioblastoma, gastrointestinal stromal tumors (GISTs) and acute myeloid leukemia through performing in vivo and in *vitro* experiments [10–12]. Through a series of preliminary pan-cancer analyses, we discovered that USP32 is significantly overexpressed in several cancer types such as BC, cholangiocarcinoma, esophageal carcinoma, head and neck squamous cell carcinoma, HCC and GC (Fig. 1A). However, among these cancer types, we noticed that high USP32 expression was significantly associated with several poorer prognostic indicators (OS, PFI and DFI) only in HCC (Figure S1). Therefore, we selected HCC as the focus of our study on the function of USP32.

In this project, we comprehensively investigated the expression, prognostic significance and functions of USP32 by employing bioinformatics analysis and several experimental assays. Notably, we also investigated the association between USP32 and the tumor microenvironment (TME) of HCC. Based on the close relationships between USP32 and immunomodulators, we further developed a USP32-derived genomic model for HCC prognosis.

## Materials and methods

### Microarray data collection

We obtained the expression of RNA sequencing in TCGA-LIHC (containing 371 HCC samples and 50 normal liver tissues) and the relevant clinical data. Gene expression data from ICGA LIRI-JP (containing 237 HCC samples) was used to verify the association between USP32 and immune infiltrates in HCC samples. In addition, the expression of USP32 in HCC and non-tumor liver tissues was also acquired from GSE36376, GSE102079, GSE164760, GSE14426 and GSE14520.

### Tissue sample collection

36 pairs of HCC samples and adjacent normal tissues were collected from diagnosed HCC patients during surgical resection in Shanghai East Hospital. After resection, these HCC tissues were immediately frozen in liquid nitrogen and stored at − 80 °C for RNA extraction.

### Tissue microarray

A human HCC tissue microarray (Cat# HStmA180Su08) that contains 75 patients’ tissues (74 are available for analysis) was purchased from Outdo Biotech, Shanghai, China. Immunohistochemical (IHC) staining for USP32 was performed using anti-USP32 antibody (1:100; sc-376,491, Santa Cruz Biotechnology). Subsequently, high- and low-USP32 expression groups were classified by estimating the number of positive cells and staining intensity of each tissue. The research and use of clinical tissues were approved by the Medical Ethics Committees of Shanghai East Hospital, Tongji University.

### Cell culture

HCC cell lines PLC/PRF/5, HepG2, HCC-LM3 and Hep3B were purchased from the Type Culture Collection of the Chinese Academy of Sciences, Shanghai, China. HCC-LM3 cells were taken from our laboratory stocks. All cell lines were authenticated by short tandem repeat (STR) profiling. Cells were cultured at 37 °C in DMEM and RPMI-1640 with 10% FBS, containing 1% penicillin and streptomycin, respectively.

### RNA isolation and quantitative real-time PCR (qRT-PCR)

The total RNA was separated from clinical samples or HCC cells through using TRIZOL reagent, and then it was reverse transcribed through utilizing TaKaRa PrimeScripttm RT Kit. The RT-qPCR reaction was carried out SYBR green Supermix (Applied Biosystems, USA) in an ABI 7500 PCR system (Applied Biosystems, USA). See Table 1 for primers for real-time PCR. The mRNA levels were normalized by β-actin.

**Table 1:** Primer sequence of USP32 and β-actin

	Forward Primer	Reverse Primer
USP32	TATACAACAGTGAGAACTACC	CCTTTTCTGTGGGAACCTTGTG
β-actin	CCTGGCACCCAGCACAATG	GGGCCGGACTCGTCATACT

### Survival analysis

Cox proportional hazards regression model was used to explore the pan-cancer relationships between the expression of USP32 and clinical prognostic indicators (OS, PFI and DFI) [13]. The Kaplan–Meier plotter was utilized to further investigate the clinical prognostic significance (OS, PFS and RFS) of USP32 in HCC patients [14]. We also explored its prognostic significance differences in different races.

### Analysis of differentially expressed genes (DEGs) between high- and low-USP32 expression groups in HCC patients

According to USP32 expression, we divided the TCGA-LIHC data into low- and high- USP32 expression groups. The R package LIMMA was utilized to analyze the DEGs between the two groups [15]. Genes met the filter criteria of P < 0.05 and |logFC| >1 were identified as DEGs [16, 17].

### Protein-protein Interaction (PPI) network construction and analysis

STRING database was employed to construct the PPI network of the DEGs between high- and low- USP32 expression groups, and Cytoscape was utilized to visualize the PPI network [18, 19]. Subsequently, we screened core modules from the PPI network through utilizing plug-in MCODE in Cytoscape [20].

### Functional enrichment analysis

We utilized Gene Set Enrichment Analysis (GSEA) analysis to examine the pathways and biological processes related to USP32 in HCC. GSEA was mainly performed with Kyoto Encyclopedia of Genes and Genomes (KEGG) [21–23].

### Estimation of tumor-microenvironment cell infiltration

The ssGSEA algorithm was utilized to explore the relative abundance of immune infiltrates in HCC samples [24]. The correlation between USP32 expression and ssGSEA scores of immune infiltrates was examined using the Spearman correlation coefficient.

### Construction and validation of a USP32-related immune prognostic model

Firstly, univariate Cox regression analyses was performed to examine if USP32-associated candidate immune genes were independent predictive markers for HCC patients’ survival. Subsequently, variables with a p-value < 0.05 were included in the multivariate Cox model to screen independent prognostic factors. After the identification of the immune-related genes, the prognostic risk scores of HCC tissues were investigated. Then, we performed Kaplan-Meier survival analysis and two-sided log-rank test through R package survival to analyze the association between risk scores and HCC patients’ survival.

### Construction of nomograms, calibration curves, and receiver operating characteristic (ROC) curves

We used the R software packages rms and survival to integrate survival time, survival status, and five USP32-related immune genes to establish a nomogram using cox methods to evaluate the prognostic significance of these USP32-related immune genes in HCC patients. Subsequently, ROC curves of 365-, 1095-, and 1825-day survival were established by using R software package pROC, and area under the curve was calculated to estimate the discrimination. Finally, calibration curves were drawn to assess the deviation of estimated probabilities from ideal values.

### Single-cell RNA-sequencing (scRNA-seq) analysis

We used the TISCH database to explore the expression of USP32 at a single-cell level in three HCC single-cell sequencing datasets GSE140228, GSE166635 and GSE98638 [25].

### Drug sensitivity analysis

We predicted drug responses of HCC patients to targeted therapeutic drugs and chemotherapy drugs based on the GDSC database [26]. pRRophetic, an R software package, was used to predict the IC50 of HCC samples through ridge regression and prediction accuracy [27].

### Western blot (WB) analysis

The procedure was performed as described in our previous study [8]. Primary antibodies used are as follows: anti-USP32 (WB: 1: 200. IHC: 1: 30, #sc-376,491, Santa Cruz Biotechnology) and anti-β-actin (1:1000, #sc-81,178, Santa Cruz Biotechnology). Immunoblots were detected by Odyssey Infrared imaging system (Li-COR Biosciences, USA) for the fluorescence method or by Chemiscope 6000 (Clinx, Shanghai, China) for the chemiluminescence method.

### RNA interference

The small interference RNAs (modified by 2′-O-methyl) against USP32 were chemically synthesized by GenePharma, Shanghai, China. The sense sequences are: siUSP32-1, 5’- GACCUGUGGACUCUCAUAUTT-3’; siUSP32-2, 5’-GCGCAUUAAAGAGGAAGAUTT-3’. The sequences for a negative control: siNC, 5’-UUCUCCGAACGUGUCACGUTT-3’. Lentiviral particles for knockdown of USP32 (shUSP32) were packaged from GenePharma, Shanghai using above corresponding sequences (siUSP32-1 and siNC) from our previous study [8]. HCC-LM3 cells were infected with the indicated lentivirus and stable cell lines were selected using puromycin.

### Cell proliferation, migration and colony formation assays

We used cell counting kit-8 (CCK8), transwell and colony formation assays to assess the proliferation, migration and colony formation abilities of HCC cells, and the procedure was performed as described in our previous study [8].

### Animal model

Twelve male BALB/c nude mice aged 4 weeks were purchased from SLAC Laboratory Animal Co., Ltd, Shanghai, China. HCC-LM3 cells were stably infected with lentivirus knocking down USP32 or its control, and subcutaneously injected into each mouse (6 mice per group). Three weeks later, the mice were sacrificed by cervical dislocation, and the tumors were collected and weighted. All animal experiments and handling procedures were approved by the Institutional Animal Care and Use Committee of Shanghai East Hospital. ARRIVE guidelines (http://arriveguidelines.org) were followed.

### Statistical analysis

R software was used for most bioinformatics analyses, and GraphPad Prism was used for graphing and statistical analysis. The statistical significance was analyzed using one-way ANOVA, *Chi*-square test, unpaired two-tailed Student’s t-test or Manne-Whitney-Wilcox test as appropriate. p < 0.05 was regarded as statistically significant.

## Results

### USP32 expression is upregulated in HCC

We firstly investigated the expression of USP32 in HCC through analyzing public databases. According to TCGA-LIHC, GSE36376, GSE102079, GSE164760, GSE14426 and GSE14520 datasets, the mRNA expression of USP32 was markedly higher in HCC samples than in non-cancer tissues (p-value < 0.0001) (Fig. 1A-G). By qRT-PCR analysis, we also discovered a marked upregulation of USP32 in HCC samples compared with normal controls (p-value < 0.01) (Fig. 1H). Finally, USP32 protein expression in a TMA was analyzed by IHC staining. The results illustrated that the protein expression level of USP32 was markedly upregulated in tumor tissues compared with normal samples (p-value < 0.0001) (Fig. 1I). All these analyses revealed that USP32 is significantly overexpressed in HCC in both mRNA and protein levels.

### Distributions of USP32 expression in clinicopathologic variables of HCC patients

**Fig. 2:**
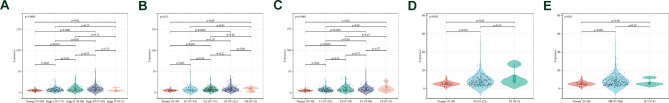
Relationship between USP32 expression and the (**A**) stage, (**B**) grade, (**C**) staged T, (**D**) staged N, (**E**) staged M in TCGA-LIHC dataset. *P < 0.05, **P < 0.01, ***P < 0.001, ****P < 0.0001. ns, No significance

A previous study has found that there are correlations of USP32 expression with clinicopathologic features in small cell lung cancer [28]. Thus, we next investigated whether there are associations between USP32 and clinicopathologic variables of HCC patients. As shown in Fig. 2, elevated USP32 expression was significantly related to the clinical stage (P < 0.0001), tumor histological grade (P < 0.01), T classification (P < 0.0001), N classification (P < 0.001), and M classification (P < 0.01) (Fig. 2A-E). Especially, higher USP32 expression was markedly found in stage II/III compared to that in stage I (Fig. 2A; P < 0.05 and 0.01, respectively), and its expression was also significantly higher in T2/3/4 compared to that in T1 (Fig. 2C; P < 0.01, 0.05 and 0.05, respectively). These analyses revealed that USP32 expression has close associations with clinicopathologic variables of HCC patients.

### High USP32 expression was an Independent predictive factor for poor outcomes in Asian HCC patients

**Fig. 3:**
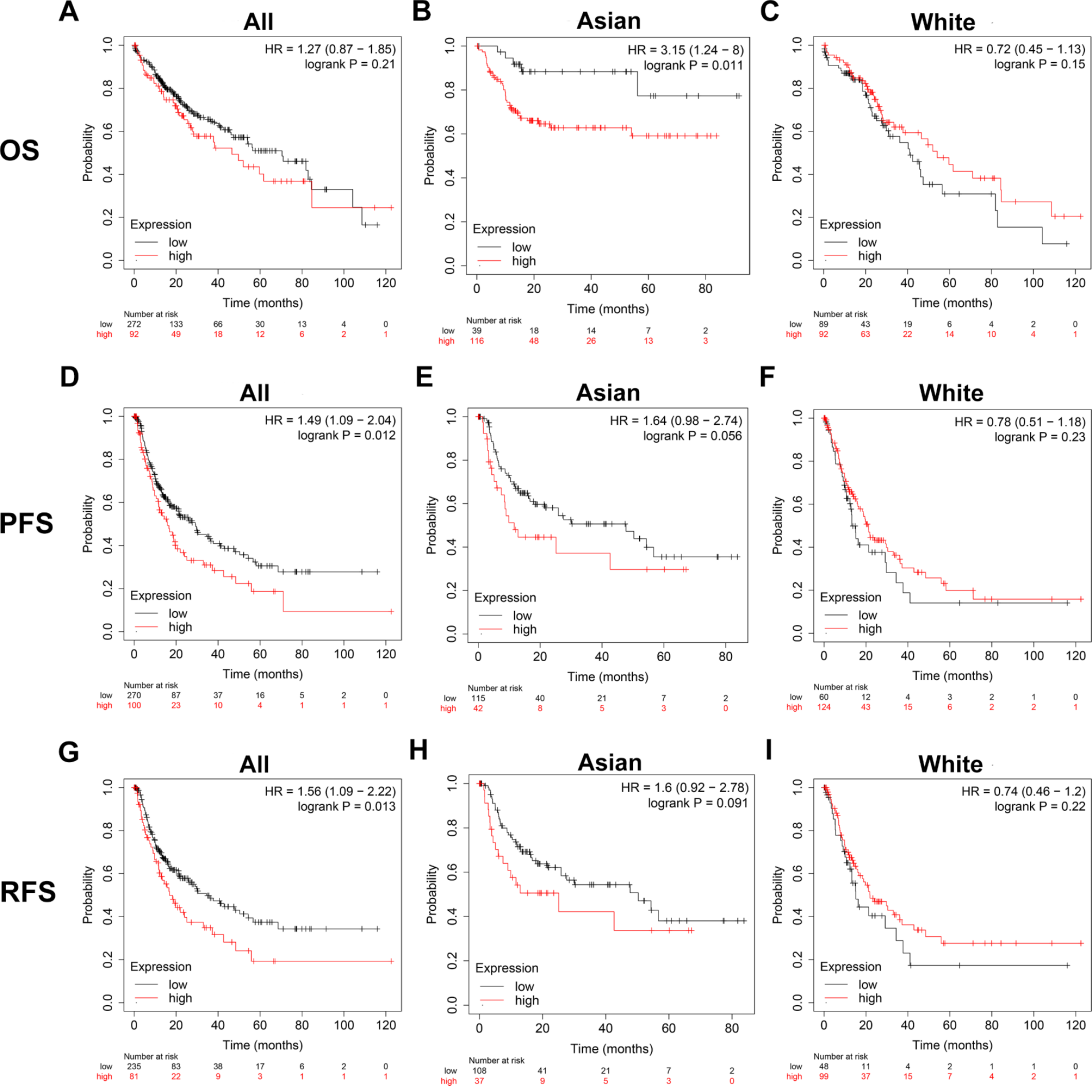
Kaplan–Meier analysis of the relationship between USP32 expression and (**A-C**) OS, (**D-F**) PFS and (**G-I**) RFS in HCC patients of different races. HR: Hazard ratios

We performed Kaplan–Meier plotter analysis aiming to explore the association between the USP32 expression level and HCC prognosis. The result illustrated that the high expression levels of USP32 were significantly associated with a shorter PFS (p = 0.012) and RFS (p = 0.013) of HCC patients, but not significantly related to OS (p = 0.21) (Fig. 3). Moreover, in analyzing Asian HCC patients, we found that high USP32 expression was significantly associated with a shorter OS (p = 0.011) and has a trend associated with a shorter PFS (p = 0.056) (Fig. 3B, E and H). However, there were no significant associations between USP32 expression and OS, PFS and RFS in White HCC patients (Fig. 3C, F and I). These results suggest that USP32 may has different prognostic significance in HCC patients of different races.

### DEGs identification and PPI network analysis revealed USP32-related molecular networks in HCC

**Fig. 4:**
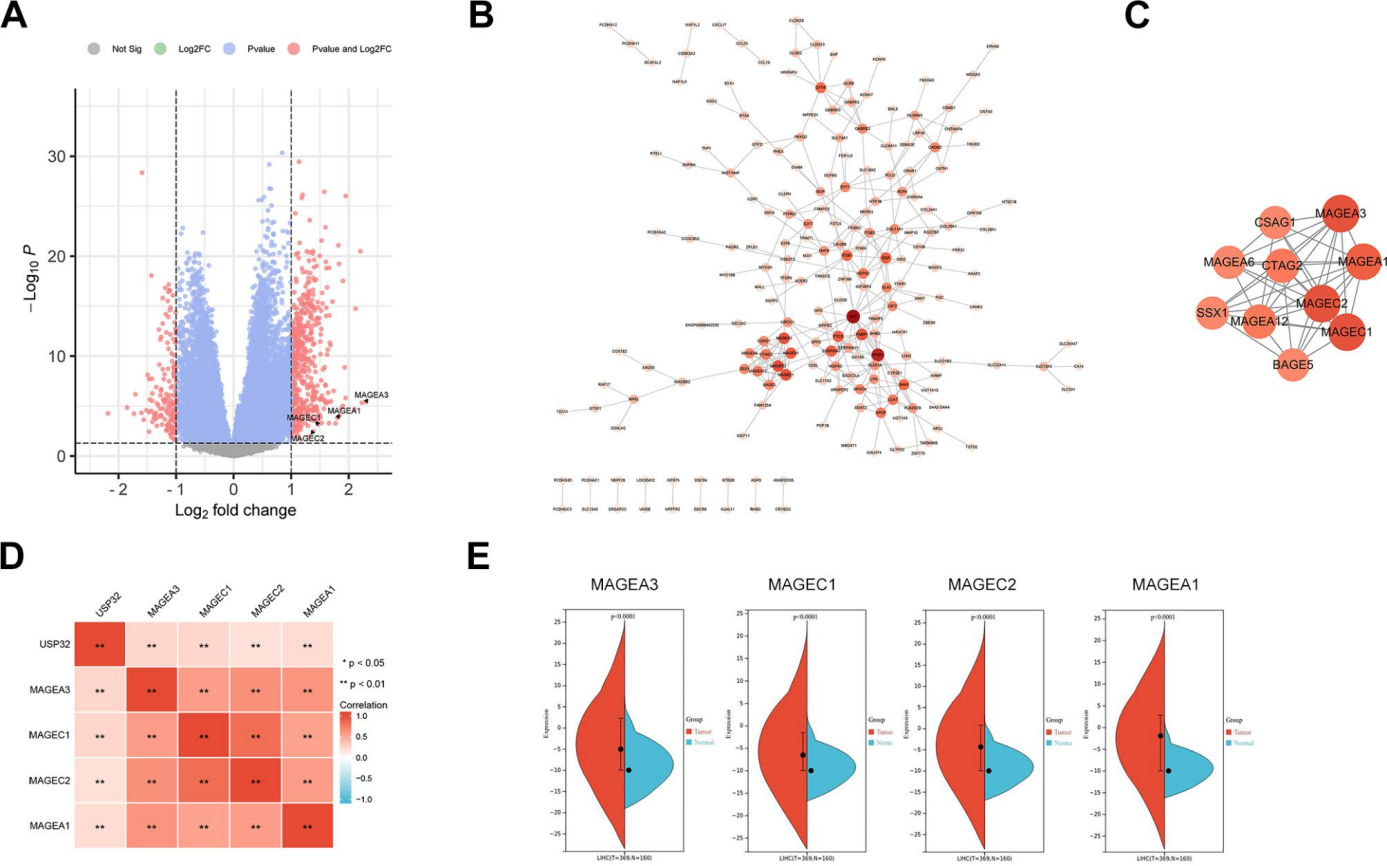
Identification of DEGs and PPI network analysis in HCC. (**A**) Volcano plot of all DEGs between high- and low-USP32 expression groups. (**B**) PPI network of all DEGs. Nodes with light color represent a higher degree of connectivity. (**C**) The most important module from the PPI network. (**D**) Correlation analysis of the expression of four key genes (MAGEA3, MAGEC1, MAGEC2 and MAGEA1) and the expression of USP32 in HCC tissues. (**E**) The expression of four key genes (MAGEA3, MAGEC1, MAGEC2 and MAGEA1) are markedly high in HCC tissues compared with normal tissues. **P < 0.01

To reveal the potential molecular networks of USP32 involvement in HCC, we firstly performed DEG analysis of 186 low-USP32 expression and 185 high-USP32 expression HCC samples from TCGA database. As a result, a total of 678 [527 (77.7%) upregulated and 151 (22.8%) downregulated] genes were identified as DEGs in the high-USP32 expression HCC group compared to the low-USP32 expression HCC group (Fig. 4A; Supplementary Table S1).

We then constructed DEGs’ PPI network that is consisted of 199 nodes (genes) and 318 interactions (Fig. 4B). Subsequently, we identified the most key module through utilizing MCODE plug-in (MCODE score = 8.667, consisting of 10 nodes and 39 interactions) (Fig. 4C). Moreover, four genes in this module with a degree ≥ 10 in the PPI network were thought to play key roles in USP32-related molecular networks in HCC, and they are all from Melanoma Antigen Gene (MAGE) family, including MAGEA3 (degree = 11), MAGEC1 (degree = 11), MAGEC2 (degree = 11) and MAGEA1 (degree = 10). Correlation analysis further revealed the significant positive correlations between these four genes and the expression of USP32 in HCC tissues (p < 0.01), and these four genes’ expression was also significantly positively related to each other (p < 0.01) (Fig. 4D). In addition, the significantly higher expression of these genes was confirmed in HCC tissues compared with normal tissues (Fig. 4E). These results indicate that USP32 may potentially regulate HCC development though regulating MAGE family members or being regulated by them.

### USP32-related signaling pathways and biological process in HCC

**Fig. 5:**
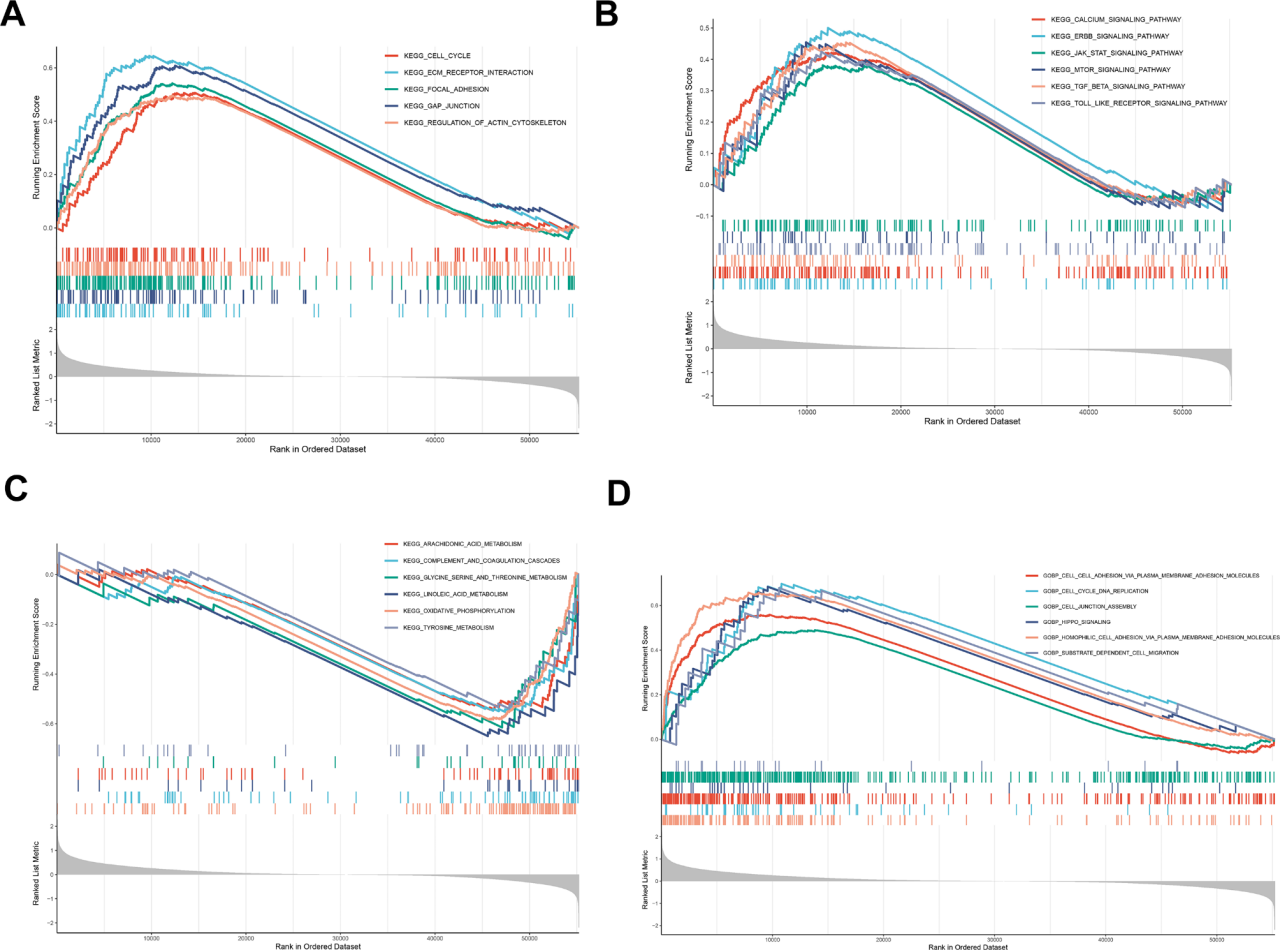
GSEA analysis of USP32 in HCC. (**A**) Cell behaviors-related KEGG items were markedly correlated with high USP32 expression. (**B**) Molecular signaling KEGG pathways were markedly correlated with high USP32 expression. (**C**) Metabolic, oxidative stress and immune-related KEGG pathways were markedly correlated with low USP32 expression. (**D**) Cell behaviors-related biological processes were markedly correlated with high USP32 expression

GSEA analysis is a crucial method for identifying the potential pathways and biological processes in which a gene or molecule may participate [29]. Thus, we performed the GSEA analysis of high- and low-USP32 expression groups. The following KEGG items were markedly enriched in HCC patients with high USP32 expression: cell behaviors-related pathways (such as ECM-receptor interaction, gap junction and focal adhesion) and molecular signaling pathways (such as ErbB, Calcium and TGF-β signaling pathways) (Fig. 5A and B). Instead, metabolic, oxidative stress and immune-related KEGG items were markedly enriched in HCC patients with low USP32 expression, such as oxidative phosphorylation, complement and coagulation cascades and linoleic acid metabolism (Fig. 5C). Finally, GO-BP functional enrichment analysis confirmed that biological processes related to cell behaviors were significantly enriched in HCC patients with high USP32 expression, such as homophilic cell adhesion, cell cycle and DNA replication, cell-cell adhesion, cell-cell junction assembly and substrate dependent-cell migration (Fig. 5D). These results revealed USP32-involved signaling pathways and biological process in HCC.

### USP32 may play a role in the TME of HCC

**Fig. 6:**
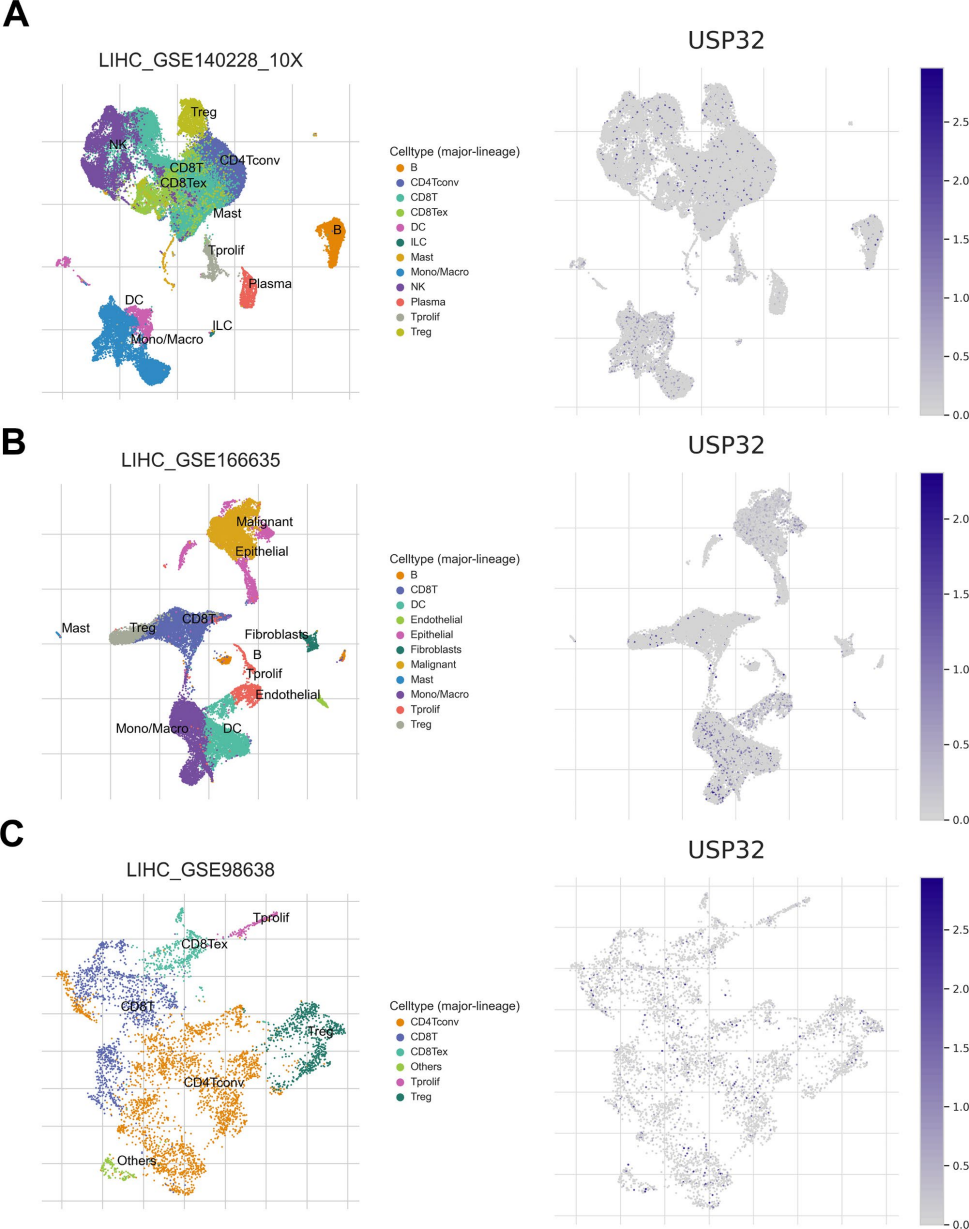
Single-cell sequencing analysis of USP32 expression in (**A**) GSE140228, (**B**) GSE166635 and (**C**) GSE98638

To explore the expression of USP32 in both malignant cells and immune cells from the TME of HCC, we analyzed three scRNA-seq databases GSE140228, GSE166635 and GSE98638. As shown in Fig. 6, USP32 expression is not only discovered in malignant cells but also in immune cells including T cells (CD4 T cells, CD8 T cells and Tregs), B cells, monocytes/macrophages, NK cells and DC cells. The widespread expression of USP32 in different kinds of immune cells confirms that it may have potential functions in the TME of HCC.

**Fig. 7:**
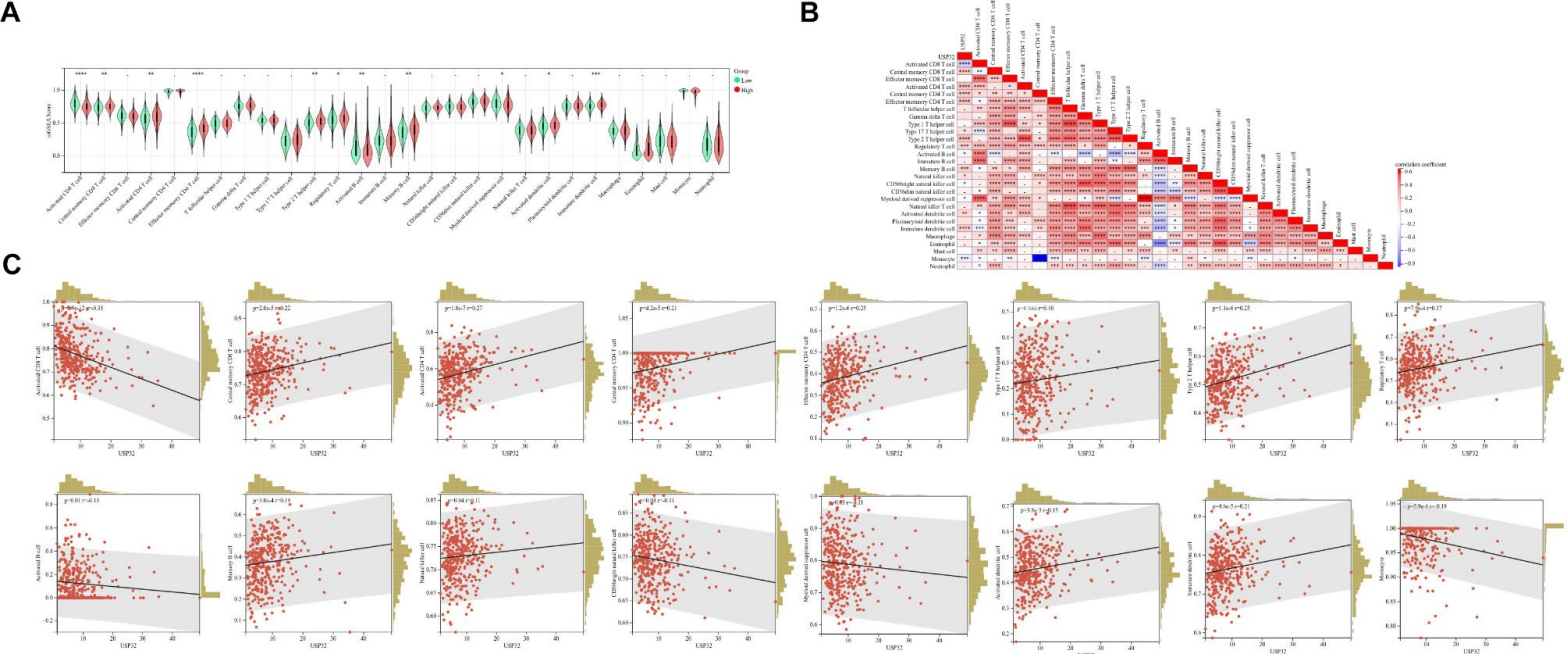
USP32 was associated with immune cell infiltration in HCC. (**A**) The levels of infiltrating immune cells between high- and low-USP32 expression groups. (**B**) The correlation of USP32 expression with infiltrating immune cells in HCC tissues. (**C**) The expression of USP32 was significantly related to the infiltration levels of immune infiltrates. *P < 0.05, **P < 0.01, ***P < 0.001, ****P < 0.0001. ns, No significance

Subsequently, we revealed that low- and high-USP32 expression groups differ in the immune infiltrates of HCC samples: central memory CD8 T cells, activated/effector memory CD4 T cells, type 2 T helper cells, regulatory T cells, memory B cells, activated and immature dendritic cells were significantly increased in the high-USP32 expressed HCC samples (P < 0.05), while the activated CD8 T cells, activated B cells and myeloid-derived suppressor cells significantly decreased (P < 0.05) (Fig. 7A). Subsequently, we confirmed and quantified the correlations between USP32 expression and infiltration levels of immune cells in HCC samples (Fig. 7B). As a result, the expression of USP32 was significantly positively correlated with the infiltration levels of activated CD4 T cells (r = 0.27), effector memory CD4 T cells (r = 0.25), type 2 T helper cells (r = 0.25), central memory CD8 T cells (r = 0.22), central memory CD4 T cells (r = 0.21), immature dendritic cells (r = 0.21), memory B cells (r = 0.19), regulatory T cells (r = 0.17), activated dendritic cells (r = 0.15), natural killer cells (r = 0.11), type 17 T helper cells (r = 0.10), and it has significant negative correlations with activated CD8 T cells (r=-0.35), monocytes (r=-0.18), activated B cells (r=-0.13), CD56bright natural killer cells (r=-0.11) and myeloid-derived suppressor cells (r=-0.11) (Fig. 7C). Finally, we used another HCC database ICGA LIRI-JP that contains 237 HCC samples, to verify the relationship between USP32 and immune infiltrates in HCC samples. As shown in Figure S2, significant correlations between USP32 expression and the infiltrations of activated CD4 T cells, type 2 T helper cells, regulatory T cells, activated CD8 T cells and monocytes were verified, which partly confirms our results.

### Construction of a USP32-derived immune model for HCC prognosis

**Fig. 8:**
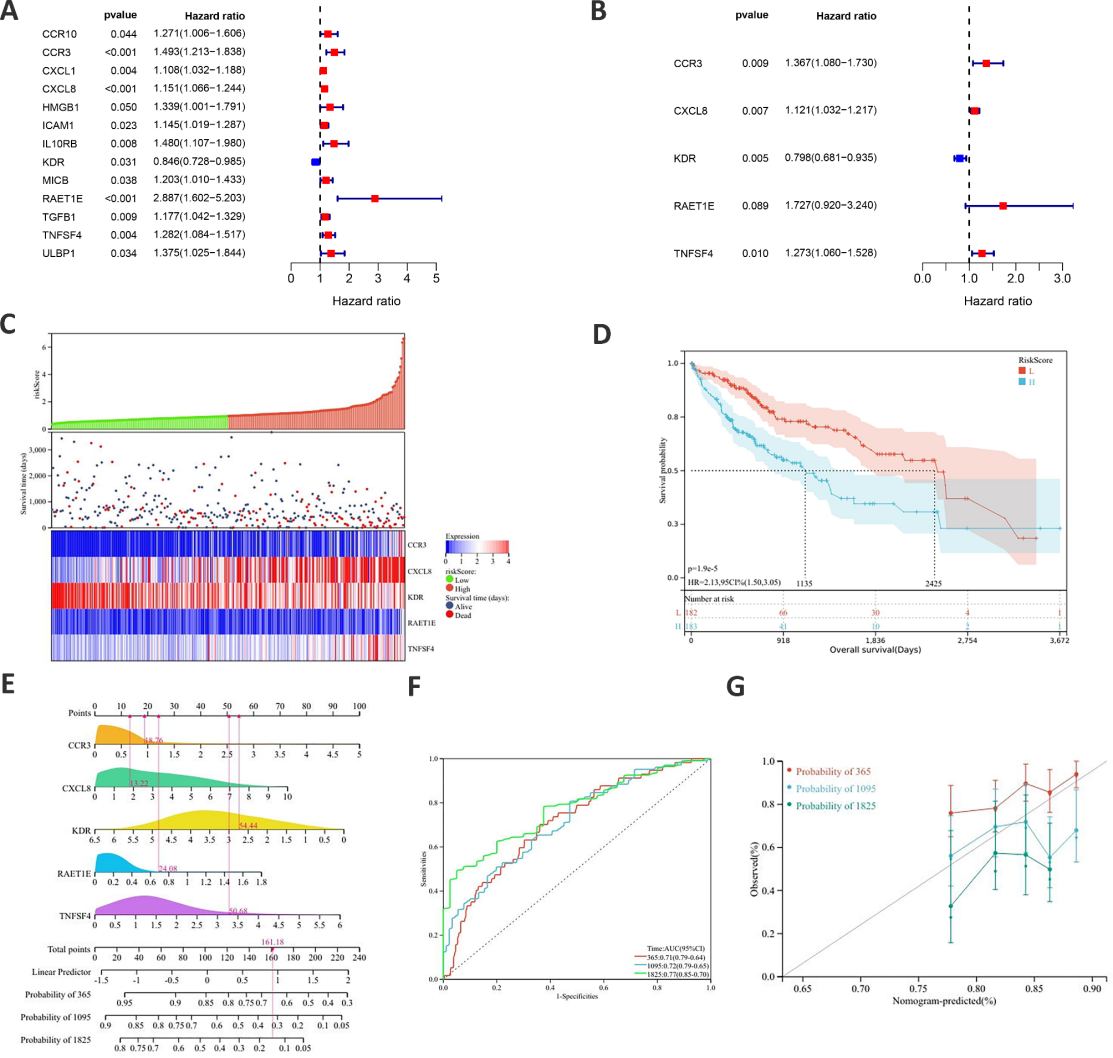
The identification of prognostic gene signatures based on 5 USP32-associated immunomodulators. (**A** and **B**) Uni- and multivariate Cox regression models are conducted to explore the association of 5 USP32-derived immunomodulators with HCC survival outcome. (**C**) Risk scores’ distribution, gene expression profiles and HCC patients’ survival statuses. (**D**) Kaplan–Meier curves for HCC patients with low or high-risk scores. (**E**) A prognostic nomogram is exploited through integrating 5 USP32-derived immunomodulators (CCR3, CXCL8, KDR, RAE1E and TNFSF4) to estimate 365-, 1095-, and 1825-day overall survival probability. (**F**) Predictive efficacy of the present nomogram by ROC curves at 365-, 1095-, and 1825-day overall survival. (**G**) Calibration plots exhibit the relationship of predicted 365-, 1095-, and 1825-day overall survival and actual survival duration

Given the potential role of USP32 in HCC immune response, we aimed to construct a USP32-related immune model for predicting HCC patient survival outcomes. Firstly, we identified 51 immunomodulators (immunostimulators such as CD40 and CD80, chemokines such as CCL7 and CXCL6, and receptors such as CCR1 and CCR4) significantly associated with the expression of USP32 in HCC samples. Secondly, we performed the univariate Cox regression analysis to estimate the potential prognostic value of USP32-associated immunomodulators in HCC. As shown in Fig. 8A and 133 immune-related genes (CCR10, CCR3, CXCL1, CXCL8, HMGB1, ICAM1, IL10RB, KDR, MICB, RAET1E, TGFB1, TNFSF4 and ULBP1) were discovered to be markedly related to the outcomes of HCC patients (Fig. 8A). Subsequently, multivariate Cox regression analysis identified and produced an optimal 5-gene (CCR3, CXCL8, KDR, RAE1E and TNFSF4) prognostic signature of HCC patients (Fig. 8B). Risk scores’ distribution, gene expression profiles and HCC patients’ survival statuses were shown in Fig. 8C. The subsequent survival analysis indicated HCC patients with low-risk scores had markedly longer survival than those with high-risk scores (P < 0.0001) (Fig. 8D).

By integrating above 5 USP32-related immunomodulators, we then exploited a nomogram to examine the survival outcomes of HCC patients (Fig. 8E). ROC curves verified the desirable efficacy of this USP32-derived risk score in predicting HCC patients’ 365-, 1095-, and 1825-day survival outcomes (Fig. 8F). In addition, the nomogram’s good prediction performance was evaluated and confirmed by calibration curves (Fig. 8G). These results demonstrated that we successfully constructed a USP32-related immune model for HCC prognosis.

### Correlation between USP32 and HCC drug sensitivity

**Fig. 9:**
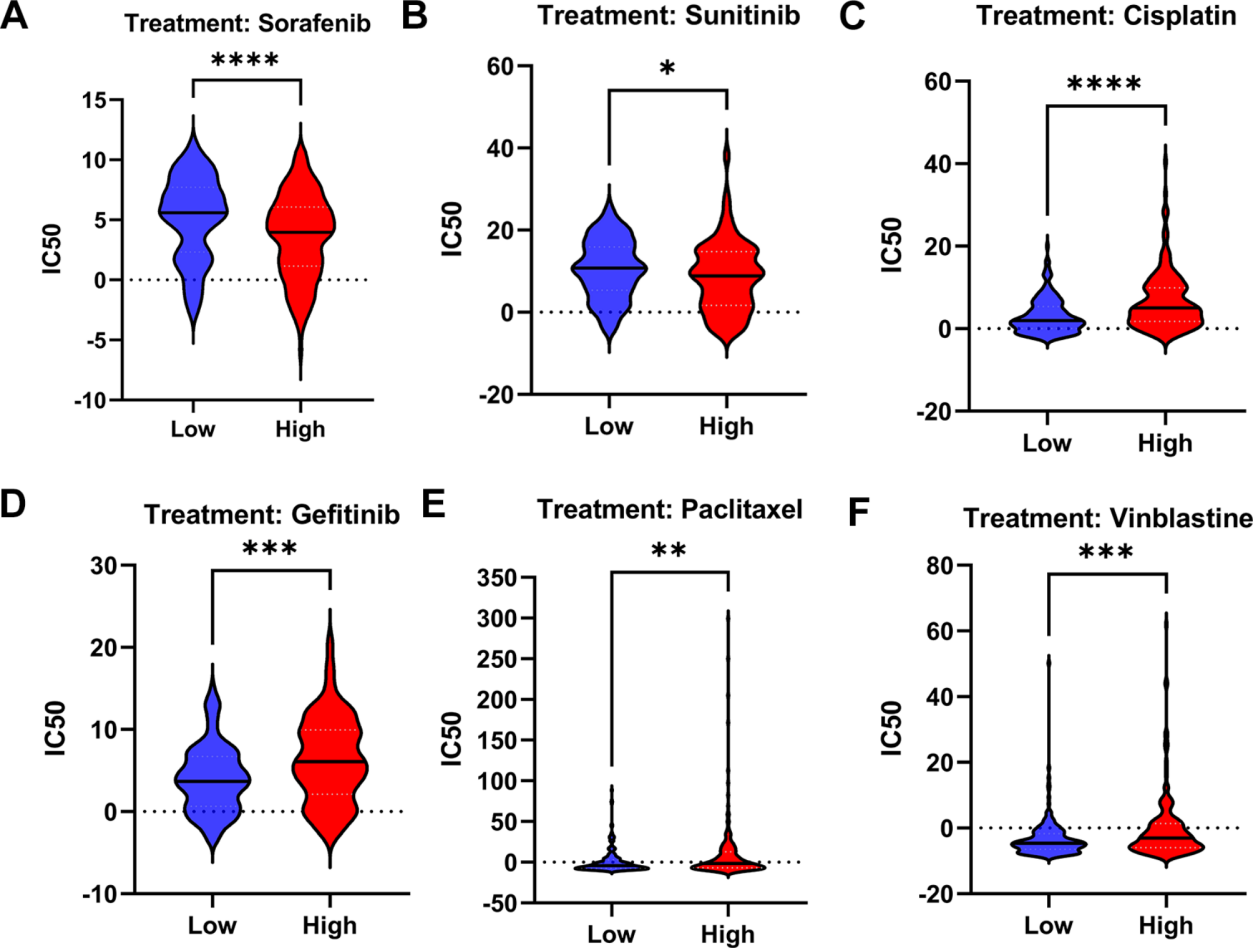
Relationship of USP32 expression with GDSC drug response. (**A-F**) Sorafenib, sunitinib, cisplatin, gefitinib, paclitaxel and vinblastine, respectively. *P < 0.05, **P < 0.01, ***P < 0.001, ****P < 0.0001

Increasing the sensitivity of tumor cells to different drugs is a key problem in the treatment of HCC [30]. According to the GDSC database, we next investigated the response of HCC individuals to targeted therapy and chemotherapy based on USP32 expression. As shown in Fig. 9, we discovered that the high-USP32 expression group exhibit markedly lower IC50 of sorafenib and sunitinib relative to the low-USP32 group, suggesting that high expression levels of USP32 presented higher sensitivity to sorafenib and sunitinib (Fig. 9A and B). In addition, the low-USP32 group showed markedly lower IC50 of cisplatin, gefitinib, paclitaxel and vinblastine, indicating that tumors with low-USP32 expression were more likely to respond to cisplatin, gefitinib, paclitaxel and vinblastine (Fig. 9C-F). These results provide a reference for researchers to identify drugs that are potentially sensitive to and resistant to high- or low-USP32 expressed HCC tumors.

### USP32 contributes to the proliferation, colony formation and migration of HCC cells in vitro and tumor growth in vivo

**Fig. 10:**
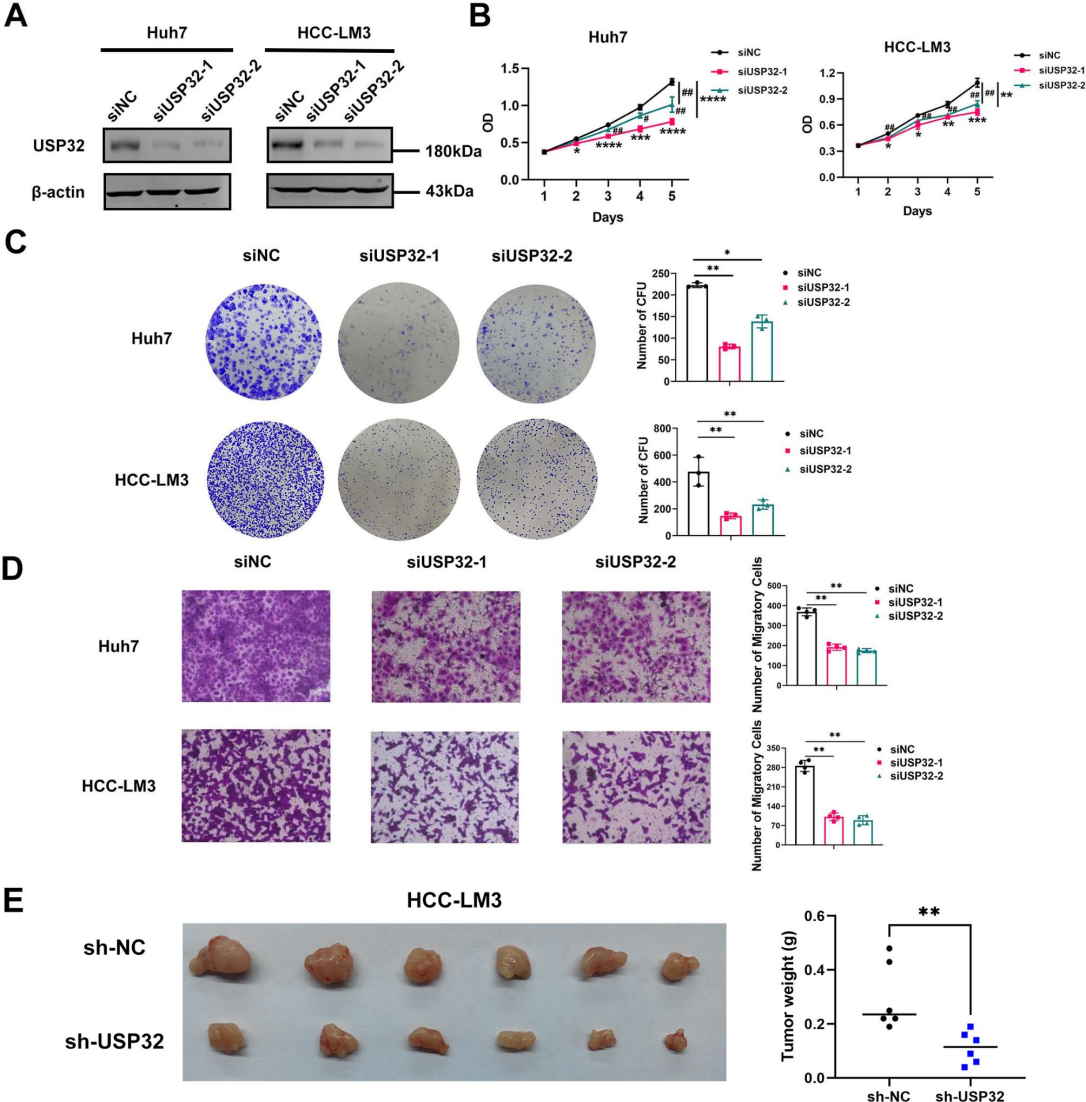
Knockdown of USP32 repressed the proliferation, colony formation and migration of HCC cells. (**A**) WB analysis exhibited USP32 expression was effectively silenced in HCC cells. Immunoblots were detected by the fluorescence method. (**B**) CCK8 assays indicated that knockdown of USP32 could markedly reduce the proliferation of HCC cells. # P < 0.05, ## P < 0.01; *P < 0.05, **P < 0.01, ***P < 0.001, **** P < 0.0001. (**C**) Colony formation assays demonstrated that inhibition of USP32 could markedly suppress the colony formation capacity of HCC cells. (**D**) The migrated cell numbers of HCC cells were significantly decreased when USP32 knockdown. (**E**) Knockdown of USP32 markedly suppressed the tumorigenicity of HCC-LM3 cells by comparing the tumor weight (n = 6 per group). All data are expressed as mean ± SD. *P < 0.05, **P < 0.01

In order to verify the oncogenic functions of USP32 in HCC, the protein expression levels of USP32 were firstly detected (Figure S3). Subsequently, two HCC cell lines Huh7 and HCC-LM3 were selected to be transfected with siRNA (2′-O-methyl modified) against USP32. WB results confirmed that USP32 expression was effectively silenced in HCC cells (Fig. 10A). Subsequently, the results of CCK8, transwell and colony formation experiments verified that knockdown of USP32 could significantly impair the proliferation, colony formation and migration of HCC cells (Fig. 10B-D). In addition to in vitro experiments, we also established a xenograft HCC model in nude mice to explore the role of USP32 in HCC progression in vivo. HCC-LM3 cells with stable downregulation of USP32 and control cells were subcutaneously injected into nude mice, and tumors were harvested three weeks after injection. As shown in Fig. 10E, tumor weight derived from the USP32-downregulation group was significantly lower than those derived from control group, verifying the oncogenic role of USP32 in the growth of HCC tumors.

## Discussion

USPs play critical roles in cancer initial, progression and metastasis. Our laboratory previously reported that USP32 promotes tumorigenesis and chemoresistance in GC [8]. In the current study, we discovered that USP32 is also overexpressed and promotes tumor progression in HCC. Notably, we found that high USP32 expression correlates with poor prognosis in Asian HCC patients but not in White HCC patients. Interestingly, distinct incidence rates and outcomes of HCC in White and Asian patients suggest that their pathogenesis is different [31–33]. Further research is required to determine whether USP32 is a specific biomarker for Asian HCC patients.

To explore potential molecular mechanisms by which USP32 regulates HCC progression, we conducted several analyses and identified key USP32-related molecular modules, including MAGEA3, MAGEC1, MAGEC2 and MAGEA1. These genes belong to the cancer-testis antigen (CTA) family and are linked to oncogenic activities such as enhanced tumor growth and EMT in HCC [34, 35]. We observed a positive correlation between USP32 expression and the expression of these CTAs, indicating a potential regulatory relationship between USP32 and CTAs.

Aberrant immune responses are closely linked to the development of cancer, and the expression of some immune-related genes can activate or suppress anti-tumor immunity, impacting the effectiveness of tumor immunotherapy [36, 37]. Through single-cell sequencing analyses, we detected USP32 expression in various immune infiltrates. Our subsequent analyses indicated a positive correlation between USP32 expression and T helper 2 cells and regulatory T cells, while it was negatively associated with activated CD8 T/B cells and CD56bright natural killer cells in HCC samples. It is noteworthy that T helper 2 cells and regulatory T cells have immunosuppressive functions, while CD8 T/B cells and CD56bright natural killer cells exhibit potent antitumor responses [38–42]. These suggest that USP32 may contribute to tumor immune escape in HCC.

Overcoming drug resistance and enhancing tumor sensitivity to drugs are crucial challenges in the treatment of HCC [43]. Recent studies have discovered that USP32 contributes to drug resistance in cancer: In several kinds of cancer cells, USP32 can confer cell resistance to a small molecule inhibitor YM155 (Sepantronium bromide) through promoting the degradation of SLC35F2, a solute-carrier protein that is essential for the uptake of YM155 [44]. In GISTs, USP32 protects Ras-related protein Rab-35 (Rab35) from proteasomal degradation, leading to tumor resistance to Imatinib, a tyrosine kinase inhibitor that is wildly used to combat GISTs [11]. In the present study, we found that HCC tumors with high USP32 expression may be more resistant to cisplatin, gefitinib, paclitaxel and vinblastine. Interestingly, a recent study also discovered that USP32 induces cisplatin resistance in GC cells [8]. Our results suggested that targeting USP32 may help to enhance the sensitivity of HCC tumors to specific drugs.

To further validate the oncogenic function of USP32, we successfully knocked down USP32 in HCC cell lines and performed functional experiments both in vivo and in vitro. However, several limitations of this project should be noted. Firstly, due to the fractions of immune infiltrates in samples from HCC patients is evaluated based on bioinformatics methods, it is critical to use human HCC samples for verifying them. However, our hospital currently lacked fresh HCC samples of sufficient quality for further TME analyses such as flow cytometry and single-cell sequencing. Thus, future studies should further confirm the correlations between USP32 and immune infiltrates in fresh human HCC samples. Secondly, some studies have reported that different TP53 status may affect the proliferative abilities of HCC cells [45, 46]. Due to the fact that Huh7 and HCC-LM3 cells carry different TP53 mutants, further studies could use HCC cell lines expressing wild-type p53 to eliminate the potential effect of TP53 status on USP32 overexpression-induced cell proliferation.

## Conclusions

In conclusion, USP32 is highly expressed in HCC and is involved in complex molecular and immunity mechanisms that regulate HCC progression. Inhibition of USP32 may potentially delay the development of HCC and improve the efficacy of chemotherapy, targeted therapy and immunotherapy.

### Electronic supplementary material

Below is the link to the electronic supplementary material.

**Table S1**. DEGs between high- and low-USP32 expression HCC groups.

Supplementary Material 2

Supplementary Material 3

Supplementary Material 4

Supplementary Material 5

## Data Availability

The datasets analysed during the current study are available in the TCGA-LIHC (https://portal.gdc.cancer.gov/projects/TCGA-LIHC), ICGA LIRI-JP (https://dcc.icgc.org/projects/LIRI-JP#!), GSE36376 (https://www.ncbi.nlm.nih.gov/geo/query/acc.cgi?acc=GSE36376), GSE102079 (https://www.ncbi.nlm.nih.gov/geo/query/acc.cgi?acc=GSE102079), GSE164760 (https://www.ncbi.nlm.nih.gov/geo/query/acc.cgi?acc=GSE164760), GSE14426 (https://www.ncbi.nlm.nih.gov/geo/query/acc.cgi?acc=GSE14426) and GSE14520 (https://www.ncbi.nlm.nih.gov/geo/query/acc.cgi?acc=GSE14520). Generated data from this study is available from the corresponding author on reasonable request.

## Abbreviations

TCGA: The Cancer Genome Atlas
GEO: Gene Expression Omnibus
TISCH: Tumor Immune Single-cell Hub
PPI: Protein-protein intertaction
GSEA: Gene Set Enrichment Analysis
ssGSEA: single sample Gene Set Enrichment Analysis
TIMER: Tumor Immune Estimation Resource
CCK8: Cell Counting Kit-8
TME: Tumor environment
OS: Overall survival, Progression-free interval
DFI: Disease-free interval
PFS: Progression-free survival
RFS: Relapse-free survival
MCODE: Molecular Complex Detection
GDSC: Genomicsof Drug Sensitivity in Cancer
DMEM: Dulbecco’s Modified Eagle Medium FBS:Fetal Bovine Serum
ECM: Extracellular matrix
DC: Dendritic cell

## References

[1] Vogel A, Meyer T, Sapisochin G, Salem R, Saborowski A (2022). Hepatocellular carcinoma. Lancet (London England).

[2] Llovet JM, Montal R, Sia D, Finn RS (2018). Molecular therapies and precision medicine for hepatocellular carcinoma. Nat Reviews Clin Oncol.

[3] Rape M (2018). Ubiquitylation at the crossroads of development and Disease. Nat Rev Mol Cell Biol.

[4] Cruz L, Soares P, Correia M. Ubiquitin-specific proteases: players in Cancer Cellular processes. Pharmaceuticals (Basel Switzerland). 2021;14(9). 10.3390/ph14090848 PMC8469789 34577547

[5] Young MJ, Hsu KC, Lin TE, Chang WC, Hung JJ (2019). The role of ubiquitin-specific peptidases in cancer progression. J Biomed Sci.

[6] Paulding CA, Ruvolo M, Haber DA (2003). The Tre2 (USP6) oncogene is a hominoid-specific gene. Proc Natl Acad Sci USA.

[7] Akhavantabasi S, Akman HB, Sapmaz A, Keller J, Petty EM, Erson AE (2010). USP32 is an active, membrane-bound ubiquitin protease overexpressed in breast cancers. Mammalian Genome: Official Journal of the International Mammalian Genome Society.

[8] Dou N, Hu Q, Li L, Wu Q, Li Y, Gao Y (2020). USP32 promotes tumorigenesis and chemoresistance in gastric carcinoma via upregulation of SMAD2. Int J Biol Sci.

[9] Nakae A, Kodama M, Okamoto T, Tokunaga M, Shimura H, Hashimoto K (2021). Ubiquitin specific peptidase 32 acts as an oncogene in epithelial Ovarian cancer by deubiquitylating farnesyl-diphosphate farnesyltransferase 1. Biochem Biophys Res Commun.

[10] Chen S, Chen X, Li Z, Mao J, Jiang W, Zhu Z (2022). Identification of ubiquitin-specific protease 32 as an oncogene in glioblastoma and the underlying mechanisms. Sci Rep.

[11] Li C, Gao Z, Cui Z, Liu Z, Bian Y, Sun H et al. Deubiquitylation of Rab35 by USP32 promotes the transmission of imatinib resistance by enhancing exosome secretion in gastrointestinal stromal tumours. Oncogene. 2023. 10.1038/s41388-023-02600-1 36725886

[12] Zhang H, Tao Y, Ding X, Wang Y, Wang X (2023). Roles of the hsa_circ_0013880/USP32/Rap1b axis in the proliferation and apoptosis of acute Myeloid Leukemia cells. Acta Biochim Biophys Sin.

[13] Andersen PK, Gill RD (1982). Cox’s regression model for counting processes: a large sample study. The Annals of Statistics.

[14] Lánczky A, Győrffy B (2021). Web-based Survival Analysis Tool tailored for Medical Research (KMplot): development and implementation. J Med Internet Res.

[15] Ritchie ME, Phipson B, Wu D, Hu Y, Law CW, Shi W (2015). Limma powers differential expression analyses for RNA-sequencing and microarray studies. Nucleic Acids Res.

[16] Zhou J, Wan R, Tian Q, Wu Z, Lin Z, Wang W (2022). Transcriptome sequencing analysis of lncRNA and mRNA expression profiles in bone Nonunion. Oxidative Med Cell Longev.

[17] Huang R, Zheng X, Wang J (2021). Bioinformatic exploration of the immune related molecular mechanism underlying pulmonary arterial Hypertension. Bioengineered.

[18] Szklarczyk D, Gable AL, Nastou KC, Lyon D, Kirsch R, Pyysalo S (2021). The STRING database in 2021: customizable protein-protein networks, and functional characterization of user-uploaded gene/measurement sets. Nucleic Acids Res.

[19] Shannon P, Markiel A, Ozier O, Baliga NS, Wang JT, Ramage D (2003). Cytoscape: a software environment for integrated models of biomolecular interaction networks. Genome Res.

[20] Bader GD, Hogue CW (2003). An automated method for finding molecular complexes in large protein interaction networks. BMC Bioinformatics.

[21] Kanehisa M, Goto S (2000). KEGG: kyoto encyclopedia of genes and genomes. Nucleic Acids Res.

[22] Kanehisa M (2019). Toward understanding the origin and evolution of cellular organisms. Protein Science: A Publication of the Protein Society.

[23] Kanehisa M, Furumichi M, Sato Y, Kawashima M, Ishiguro-Watanabe M (2023). KEGG for taxonomy-based analysis of pathways and genomes. Nucleic Acids Res.

[24] Hänzelmann S, Castelo R, Guinney J (2013). GSVA: gene set variation analysis for microarray and RNA-seq data. BMC Bioinformatics.

[25] Sun D, Wang J, Han Y, Dong X, Ge J, Zheng R (2021). TISCH: a comprehensive web resource enabling interactive single-cell transcriptome visualization of Tumor microenvironment. Nucleic Acids Res.

[26] Yang W, Soares J, Greninger P, Edelman EJ, Lightfoot H, Forbes S (2013). Genomics of Drug Sensitivity in Cancer (GDSC): a resource for therapeutic biomarker discovery in cancer cells. Nucleic Acids Res.

[27] Geeleher P, Cox N, Huang RS (2014). pRRophetic: an R package for prediction of clinical chemotherapeutic response from Tumor gene expression levels. PLoS ONE.

[28] Hu W, Wei H, Li K, Li P, Lin J, Feng R. Downregulation of USP32 inhibits cell proliferation, migration and invasion in human small cell Lung cancer. Cell Prolif. 2017;50(4). 10.1111/cpr.12343 PMC6529138 28597490

[29] Subramanian A, Tamayo P, Mootha VK, Mukherjee S, Ebert BL, Gillette MA (2005). Gene set enrichment analysis: a knowledge-based approach for interpreting genome-wide expression profiles. Proc Natl Acad Sci USA.

[30] Marin JJG, Macias RIR, Monte MJ, Romero MR, Asensio M, Sanchez-Martin A et al. Molecular bases of Drug Resistance in Hepatocellular Carcinoma. Cancers. 2020;12(6). 10.3390/cancers12061663 PMC7352164 32585893

[31] Pham C, Fong TL, Zhang J, Liu L (2018). Striking Racial/Ethnic disparities in Liver Cancer incidence rates and temporal trends in California, 1988–2012. J Natl Cancer Inst.

[32] Hoehn RS, Hanseman DJ, Wima K, Ertel AE, Paquette IM, Abbott DE (2015). Does race affect management and survival in hepatocellular carcinoma in the United States?. Surgery.

[33] Wen GM, Song CL, Liu DH, Xia P (2023). Different races have different immune microenvironments: comparison of White and Asian patients with Liver cancer. Am J cancer Res.

[34] Craig AJ, Garcia-Lezana T, Ruiz de Galarreta M, Villacorta-Martin C, Kozlova EG, Martins-Filho SN (2021). Transcriptomic characterization of cancer-testis antigens identifies MAGEA3 as a driver of Tumor progression in hepatocellular carcinoma. PLoS Genet.

[35] Gu X, Mao Y, Shi C, Ye W, Hou N, Xu L (2019). MAGEC2 correlates with unfavorable prognosis and promotes Tumor Development in HCC Via epithelial-mesenchymal transition. OncoTargets and Therapy.

[36] Chen DS, Mellman I (2017). Elements of cancer immunity and the cancer-immune set point. Nature.

[37] Spranger S, Gajewski TF (2018). Impact of oncogenic pathways on evasion of antitumour immune responses. Nat Rev Cancer.

[38] Chen Y, Sun J, Luo Y, Liu J, Wang X, Feng R (2022). Pharmaceutical targeting Th2-mediated immunity enhances immunotherapy response in Breast cancer. J Translational Med.

[39] Dees S, Ganesan R, Singh S, Grewal IS (2021). Regulatory T cell targeting in cancer: emerging strategies in immunotherapy. Eur J Immunol.

[40] Philip M, Schietinger A (2022). CD8(+) T cell differentiation and dysfunction in cancer. Nat Rev Immunol.

[41] Wang SS, Liu W, Ly D, Xu H, Qu L, Zhang L (2019). Tumor-infiltrating B cells: their role and application in anti-tumor immunity in Lung cancer. Cell Mol Immunol.

[42] Wagner JA, Rosario M, Romee R, Berrien-Elliott MM, Schneider SE, Leong JW (2017). CD56bright NK cells exhibit potent antitumor responses following IL-15 priming. J Clin Investig.

[43] Bao MH, Wong CC, Hypoxia. Metabolic reprogramming, and Drug Resistance in Liver Cancer. Cells. 2021;10(7). 10.3390/cells10071715 PMC8304710 34359884

[44] Chandrasekaran AP, Kaushal K, Park CH, Kim KS, Ramakrishna S (2021). USP32 confers cancer cell resistance to YM155 via promoting ER-associated degradation of solute carrier protein SLC35F2. Theranostics.

[45] Zhou CC, Yang F, Yuan SX, Ma JZ, Liu F, Yuan JH (2016). Systemic genome screening identifies the outcome associated focal loss of long noncoding RNA PRAL in hepatocellular carcinoma. Hepatology (Baltimore MD).

[46] Tang W, Xue R, Weng S, Wu J, Fang Y, Wang Y (2015). BIRC6 promotes hepatocellular carcinogenesis: interaction of BIRC6 with p53 facilitating p53 degradation. Int J Cancer.

