# Supplementary figures and images for: High USP32 expression contributes to cancer progression and is correlated with immune infiltrates in hepatocellular carcinoma
Source: BMC Cancer. 2023 Nov 13;23:1105. doi: 10.1186/s12885-023-11617-4 (PMC10644423; doi:10.1186/s12885-023-11617-4)

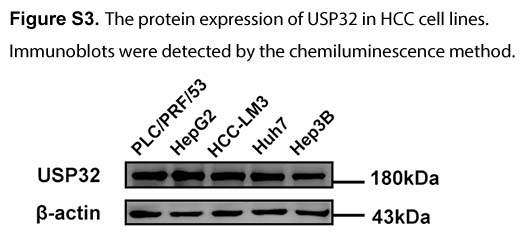

Supplement: Supplementary file 2 — Supplementary Material 2 [file 12885_2023_11617_MOESM2_ESM.jpg]

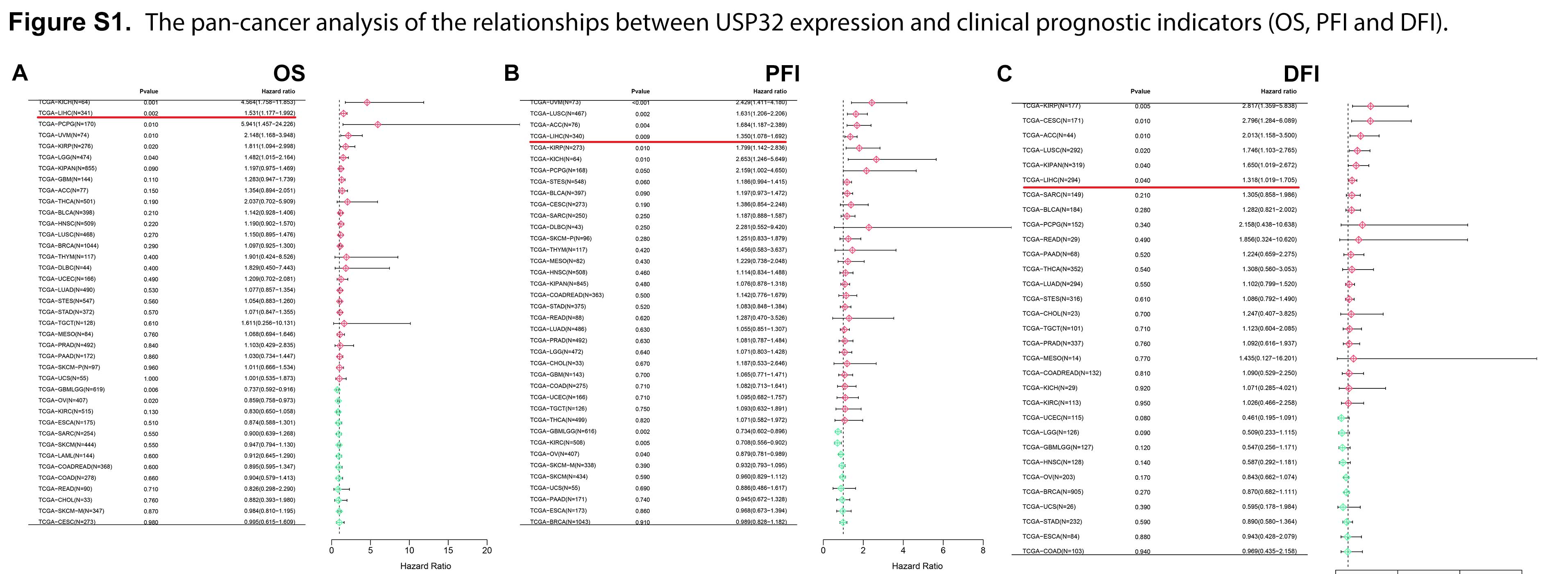

Supplement: Supplementary file 3 — Supplementary Material 3 [file 12885_2023_11617_MOESM3_ESM.jpg]

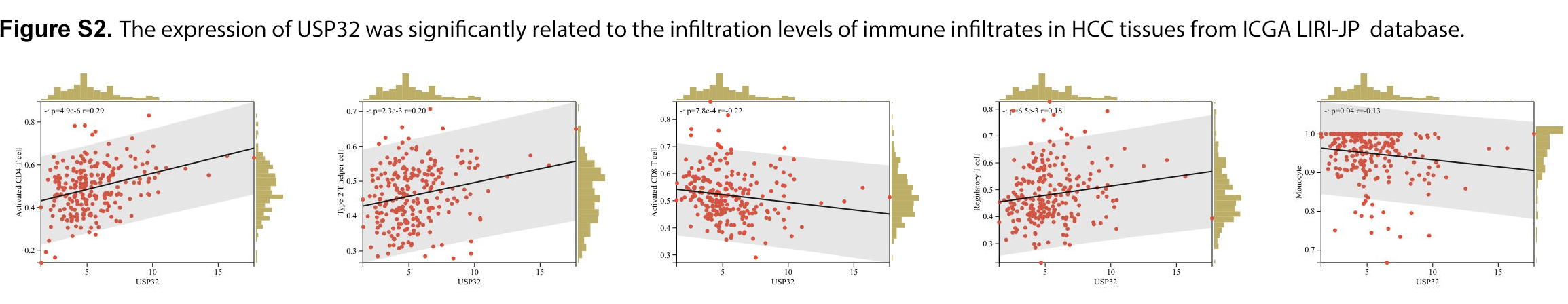

Supplement: Supplementary file 4 — Supplementary Material 4 [file 12885_2023_11617_MOESM4_ESM.png]

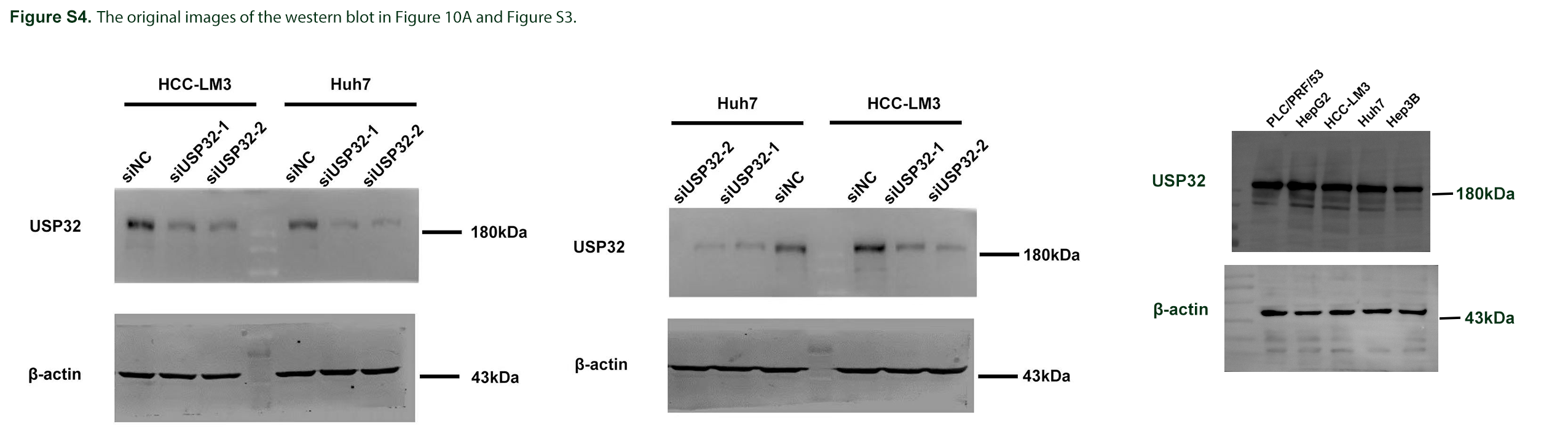

Supplement: Supplementary file 5 — Supplementary Material 5 [file 12885_2023_11617_MOESM5_ESM.png]
